# Bacterial Sirtuins Overview: An Open Niche to Explore

**DOI:** 10.3389/fmicb.2021.744416

**Published:** 2021-10-26

**Authors:** Julia Gallego-Jara, Álvaro Ortega, Gema Lozano Terol, Rosa A. Sola Martínez, Manuel Cánovas Díaz, Teresa de Diego Puente

**Affiliations:** Department of Biochemistry and Molecular Biology (B) and Immunology, Faculty of Chemistry, University of Murcia, Campus de Espinardo, Regional Campus of International Excellence “Campus Mare Nostrum”, Murcia, Spain

**Keywords:** sirtuins, bacteria, deacetylation, metabolism, prokaryote

## Abstract

Sirtuins are deacetylase enzymes widely distributed in all domains of life. Although for decades they have been related only to histones deacetylation in eukaryotic organisms, today they are considered global regulators in both prokaryotes and eukaryotes. Despite the important role of sirtuins in humans, the knowledge about bacterial sirtuins is still limited. Several proteomics studies have shown that bacterial sirtuins deacetylate a large number of lysines *in vivo*, although the effect that this deacetylation causes in most of them remains unknown. To date, only the regulation of a few bacterial sirtuin substrates has been characterized, being their metabolic roles widely distributed: carbon and nitrogen metabolism, DNA transcription, protein translation, or virulence. One of the most current topics on acetylation and deacetylation focuses on studying stoichiometry using quantitative LC-MS/MS. The results suggest that prokaryotic sirtuins deacetylate at low stoichiometry sites, although more studies are needed to know if it is a common characteristic of bacterial sirtuins and its biological significance. Unlike eukaryotic organisms, bacteria usually have one or few sirtuins, which have been reported to have closer phylogenetic similarity with the human Sirt5 than with any other human sirtuin. In this work, in addition to carrying out an in-depth review of the role of bacterial sirtuins in their physiology, a phylogenetic study has been performed that reveals the evolutionary differences between sirtuins of different bacterial species and even between homologous sirtuins.

## Lysine Acetylation Is a Global Post-Translational Modification

Protein acetylation is a post-translational modification (PTM) consisting of the transfer of an acetyl group from an acetyl donor to a protein residue. Two different types of protein acetylation have been identified, *N*-α-acetylation or terminal acetylation and *N*-ε-acetylation or lysine acetylation (Drazic et al., 2016). In *N*-α-acetylation, the transfer of an acetyl group from acetyl-CoA to an N-terminal residue of a peptide or protein is catalyzed by *N*-acetyltransferases (NATs) (Drazic et al., 2016). N-terminal acetylation is frequent in eukaryotic organisms, although it is proposed to be less common in bacteria (Nguyen et al., 2018; Parks and Escalante-Semerena, 2020). In contrast, *N*-ε-acetylation is a PTM widely distributed in all domains of life (Wagner and Payne, 2013; Macek et al., 2019). *N*-ε-acetylation consists of the transfer of an acetyl group from acetyl-CoA or acetyl-phosphate to the *N*-ε group of a lysine residue of a protein. Moreover, unlike N-terminal acetylation, *N*-ε-acetylation can occur without the participation of any enzyme (Wagner and Payne, 2013; Kuhn et al., 2014; James et al., 2017). *N*-ε-acetylation was for decades only related to histone acetylation by histone acetyltransferases (HATs) (Hebbes et al., 1988). However, advances in mass spectrometry allowed the identification of many non-histone acetylated proteins in the three domains of life (Spange et al., 2009; Zhang et al., 2009). Due to the wide diversity of acetylated proteins, histone acetyltransferases were renamed as lysine acetyltransferases (KATs). Regarding *N*-ε-acetylation in bacteria, the first bacterial lysine acetylome was reported in *E. coli* in 2008. Subsequently, several acetylome studies have been reported across bacterial species (Christensen et al., 2019). These studies have shown that protein acetylation is one of the most frequent PTM, highly conserved and ancient in prokaryotes (Yu et al., 2008; Crosby et al., 2012; Wua et al., 2013; Castaño-Cerezo et al., 2014; Kosono et al., 2015; Ouidir et al., 2015; Xie et al., 2015; Nakayasu et al., 2017; Wei et al., 2017; Li et al., 2018).

Bacterial acetylation has become an important mechanism to regulate metabolism and virulence allowing organisms their adaptation to different environments (Bernal et al., 2014; Nurulain and Zaveri, 2016; Carabetta and Cristea, 2017; Macek et al., 2019). Acetylation can occur by two different mechanisms, catalyzed by KATs or through a non-enzymatic mechanism. Non-enzymatic lysine acetylation is positioned as the most frequent mechanism of acetylation in the prokaryotic model *Escherichia coli* (*E. coli*). Furthermore, lysines that are acetylated by a KAT are not usually non-enzymatically acetylated, and vice versa (Weinert et al., 2013a; Kuhn et al., 2014; Christensen et al., 2018). The importance of lysine acetylation in metabolism regulation depends, in part, on its reversibility. Acetylation of proteins can be reverted by lysine deacetylases (KDACs). Depending on their sequence and domain organization, KDACs have been divided into four groups: class I, II, and IV KDACs need Zn^2+^, while class III, also known as Sir-2 like proteins or sirtuins, need oxidized nicotinamide adenine dinucleotide (NAD^+^) to carry out protein deacetylation (Frye, 2000; Gregoretti et al., 2004). Interestingly, a proteomic study carried out by AbouElfetouh et al. (2015) demonstrated that in *E. coli*, CobB sirtuin can deacetylate lysines both enzymatically and non-enzymatically acetylated, without a clear preference. Furthermore, this study identified 69 lysines reproducibly, significantly, and robustly more acetylated in a deficient *cobB* strain than in wild type. Nevertheless, most non-enzymatic acetylation events do not appear to be reversed by a KDAC (Weinert et al., 2013a; Kuhn et al., 2014; AbouElfetouh et al., 2015).

Beyond the identification of acetylation and deacetylation points in proteins and acetylation fold-changes under different conditions or mutants, precise measures of acetylation site occupancy, or stoichiometry, were not possible until a few years ago. In recent years the development of quantitative LC-MS/MS methods has allowed the study of the stoichiometry of acetylation and deacetylation (Baeza et al., 2014; Meyer et al., 2016; Gil et al., 2017; Weinert et al., 2017). Acetylation or deacetylation stoichiometry of a modificated lysine refers to the fraction of that lysine that is acetylated or deacetylated. To our knowledge, two works have studied how the deletion of *E. coli cobB* gene affects the acetylation stoichiometry, concluding that the absence of CobB increases acetylation at low stoichiometry sites, which had also been demonstrated for human Sirt3 (Baeza et al., 2014; Weinert et al., 2015, 2017). This result and the similarities between CobB and SIRT3 suggest an evolutionary origin of sirtuin deacetylases and a promiscuous activity, which needs to be further investigated to know if it is a common characteristic of all sirtuins. The fact that deacetylation occurs at very low stoichiometries could indicate a low impact of this modification on overall activity, however, it is not necessarily true, because deacetylation may be carried out on a small fraction of proteins that are spatially or temporally different from the rest (Olsen and Mann, 2013). The stoichiometry of sirtuin-mediated deacetylation is essential to know its real physiological role, and how it changes according to environmental conditions. Despite the advances made in recent years in this field, it is necessary to continue the study of the stoichiometry of acetylation and deacetylation in other organisms, both prokaryotic and eukaryotic.

## Sirtuins: A Regulatory Family of Proteins Conserved Since Bacteria to Humans

Sirtuins are lysine deacylase enzymes consuming NAD^+^ and producing nicotinamide (NAM) and 2′-*O*-acetyl-ADP-ribose. In the 1970s, the first sirtuin was identified in *Saccharomyces cerevisiae* (*S. cerevisiae*) and was named Sir2 (Silent informator regulator 2) (Klar et al., 1979; Ivy et al., 1986). However, its essential role on replicative lifespan and deacetylase activity was not discovered until much later (Kennedy et al., 1995; Kaeberlein et al., 1999; Imai et al., 2000). Since the discovery of Sir2, several Sir2-like proteins were identified in prokaryotes and eukaryotes organisms, and Sir2-like proteins became known as sirtuins (Brachmann et al., 1995). The role of sirtuins in eukaryotic organisms has been widely studied and, to date, seven Sir2 like deacetylases (Sirt1-Sirt7) have been identified in human cells.

Sirtuins share an approximately 275 amino acid long conserved core and a variable N- and C-terminal domains (Costantini et al., 2013). In the conserved core exists a NAD^+^ binding domain consisting in a Rossmann fold and a Zn^2+^ binding domain coordinated with four Cys residues in a tetrahedral geometry. These two domains are linked by a loop area where the acetylated peptide is accommodated. Regarding the area of substrate binding to the catalytic domain, sirtuins show three pockets: (A) where the ADP-ribose intermediate binds, (B) where the nicotinamide-ribose interaction occurs, and (C) where the nicotinamide is located, allowing the transfer of the acetyl group from lysine to ribose (Avalos et al., 2002; Yuan and Marmorstein, 2012). Beyond the central core conserved in all sirtuins, the length and structure of the terminal amino and carboxyl are highly variable, with several intrinsically disordered structures that are proposed to be involved in the determination of the specificity and subcellular location of the sirtuin (Costantini et al., 2013).

The first bacterial sirtuin was identified in *Salmonella enterica (S. enterica)* and was called CobB because it was related to cobalamin biosynthesis and propionic catabolism (Tsang and Escalante-Semerena, 1996; Tsang and Escalante-semerena, 1998). Shortly after, deacetylation of acetyl-CoA synthetase (Acs) in *S. enterica* by CobB was characterized (Starai et al., 2002). This discovery meant a turning point in sirtuin research: the regulation by N-ε deacetylation of a non-histone protein was characterized for the first time. Shortly after this first identification in *Salmonella*, a bacterial sirtuin, CobB from *E. coli*, was crystallized and its structure solved, concluding that cognate substrate binding involves the Zn-binding domain and regions distal to the acetyl-lysine pocket (Zhao et al., 2004).

*Escherichia coli* CobB was identified as the first prokaryotic desuccinylase enzyme (Colak et al., 2013). Succinylation, previously identified in mitochondria, has been described as a frequent modification in prokaryotes, overlapping extensively with acetylation (Du et al., 2011; Weinert et al., 2013b). In *Bacillus subtilis* (*B. subtilis*), proteomic studies have identified hundreds of acetylated and succinylated proteins. The increase in the acetylation and succinylation observed in the two *B. subtilis* deacetylase deficient strains, Δ*acuC*Δ*srtN*, suggests a role of both of them in the global deacetylation and desuccinylation systems (Kim et al., 2013; Kosono et al., 2015). In this sense, a recent study carried out a kinetic characterization of SrtN over different acylated substrates concluding that this sirtuin catalyzes efficiently other deacylations different to acetylation, such as malonylation or formylation (Seidel et al., 2016). Propionylation has also been reported to play a vital role in cellular physiology of prokaryotes and eukaryotes. It is a reversible PTM, depropionylation being catalyzed by sirtuins as human Sirt1 or CobB from *E. coli* and *S. enterica* (Garrity et al., 2007; Cheng et al., 2009; Sun et al., 2016b). In addition, some sirtuins, like the *Lactobacillus acidophilus* (*L. acidophilus*) Sir2La, the unique characterized sirtuin from *L. acidophilus*, shows a preference for propionylated over acetylated and butyrylated substrates (Olesen et al., 2018).

Beyond “classical” deacylations, sirtuins are related to other deacylation reactions such as delipoamidation or dehomocysteinylation. Lipoamidation is a modification identified in eukaryotes in the 1950s responsible for the regulation of multimeric enzyme complexes. However, it has been poorly studied in prokaryotes (Reed et al., 1958; Reed, 2001). Reversible lipoamidation has been identified in eukaryotes and prokaryotes, being CobB the first bacterial sirtuin with delipoamidase activity (Mathias et al., 2014; Rowland et al., 2017). In *E. coli*, CobB regulates pyruvate dehydrogenase (PDH) and ketoglutarate dehydrogenase (KDH) complexes, two carbon entry points into the tricarboxylic acid cycle, by delipoamidation, which highlights the role in central metabolism of this sirtuin. Lysine dehomocysteinylation activity has been attributed to *S. enterica* CobB, the only prokaryotic enzyme with this activity to date. This interesting finding suggests that sirtuins might play a role in hyperhomocysteinemia, the increase of homocysteinylated proteins in human which is related to many diseases (Mei et al., 2016). In addition to these deacylase activities, some sirtuins also catalyze ADP ribosylations, a modification consisting in the transfer of the ADP-ribose group from NAD^+^ to an acceptor, producing mono-ADP-ribosylated proteins and nicotinamide (Appel et al., 2016). In bacteria, auto ADP-ribosylation of a sirtuin from *Mycobacterium smegmatis* (*M. smegmatis*) has been identified as essential for growth in natural environments (Tan et al., 2015). ADP-ribosylation activity has been also associated to sirtuins from microbial pathogens (Rack et al., 2015).

## Sirtuins in Carbon and Nitrogen Metabolism

Deacetylation of acetylated Acs K609 by CobB in *S. enterica* (also known SeCobB) was the first described bacterial sirtuin reaction (Starai et al., 2002, 2003). Following this milestone, Acs protein has been shown to be a substrate for other sirtuins in many prokaryotic and eukaryotic organisms (Schwer et al., 2006; Gardner and Escalante-Semerena, 2009; Crosby et al., 2010; Xu et al., 2011; Mikulik et al., 2012; Hayden et al., 2013; Tucker and Escalante-Semerena, 2013; De Diego Puente et al., 2015; VanDrisse and Escalante-Semerena, 2018; Burckhardt et al., 2019). A recent study has shown that the binding of cAMP to Acs inhibits its deacetylation by SeCobB (Han et al., 2017). Our group carried out the kinetic characterization of *E. coli* Acs deacetylation by CobB, showing that deacetylated a minimum of 20 acetylated lysines of Acs with a single kinetic rate, which suggests that the main determinants of sirtuin specificity are the structural protein components rather than the protein sequence. This is, to our knowledge, the only sirtuin kinetic characterization with a fully acetylated and natively folded protein as a substrate (Gallego-Jara et al., 2017). Acs from *B. subtilis*, AcsA, is essential to consume the acetate present in soil, the natural habitat of this bacterium (Grundy et al., 1993). The activity of *B. subtilis* AcsA has been reported to be regulated by acetylation/deacetylation. However, while acetylation is catalyzed by AcuA acetyltransferase, two lysine deacetylases are involved in its deacetylation: NAD^+^ independent AcuC and sirtuin SrtN (Gardner et al., 2006; Gardner and Escalante-Semerena, 2009).

*Rhodopseudomonas palustris* (*R. palustris*) is a photoheterotrophic bacterium able to degrade aromatic compounds to acetyl-CoA, which makes it an interesting weapon against environmental contamination by human-produced aromatic compounds. In *R. palustris*, reversible acetylation regulates acyl-CoA synthetases responsible for aromatic activation. Interestingly, activation of three of these acyl-CoA synthetases (Benzoyl-CoA (BadA), 4-hydroxybenzoyl-CoA (HbaA) and cyclohexanecarboxyl-CoA (AliA) synthases) through lysine deacetylation can be catalyzed by the sirtuin RpSrtN or by the Zn-dependent protein deacetylase LdaA (Crosby et al., 2010, 2012).

In 2007, Garrity et al. demonstrated that expression of CobB in *S. enterica* was necessary for it to grow with acetate or propionate as the only carbon source. The CobB activity need for growth in propionate triggered the identification of the enzyme PrpE (propionyl-CoA synthetase) as a new substrate of this sirtuin. In this case, SeCobB catalyzed the depropionylation of the 592 PrpE lysine, which induced its activation. This study was the first to reveal an activity different to deacetylation in a bacterial sirtuin (Garrity et al., 2007). In *Mycobacterium tuberculosis* and *M. smegmatis* reversible acetylation is also involved in propionate metabolism through acetylation and deacetylation carried out by the Rv1151c sirtuin or the ortholog in *M. smegmatis*, SrtN (Hayden et al., 2013; Nambi et al., 2013). Interestingly, while Rv1151c is the only found Sir2 like protein in *M. tuberculosis*, two predicted sirtuins, Ms5175 and Ms4620, are observed in *M. smegmatis*, showing Ms4620 a robust ADP-ribosylation activity (Tan et al., 2015). *M. tuberculosis* fatty acyl CoA synthetases (FadD22, FadD2, FadD5, and FadD13), AMP-forming acyl-CoA synthetases similar to Acs, are also acetylated and deacetylated by Rv1151. This modification activates fatty acids metabolism (Nambi et al., 2013). Further, FadD33, a *M. smegmatis* fatty acid synthetase that is indispensable for *Mycobacterium* virulence development through siderophores, is also regulated by acetylation and deacetylation through its SrtN sirtuin (Vergnolle et al., 2013).

Beyond acyl-CoA synthetases, other metabolic enzymes have been reported to be regulated by acetylation/deacetylation. For example, the *S. enterica*’s glyceraldehyde-3-phosphate dehydrogenase (GapA), isocitrate lyase (Icl), or isocitrate dehydrogenase phosphatase/kinase (AceK) enzymes (Wang et al., 2010). Results from this study were questioned however in other reports (Crosby et al., 2012; Crosby and Escalante-Semerena, 2014). Isocitrate lyase from *M. tuberculosis* is also regulated by sirtuin deacetylation (Bi et al., 2017). The first substrate of CobB identified in *E. coli* was the chemotactic regulator CheY (Li et al., 2010). CheY is an essential regulator of chemotaxis, the mechanism through which bacteria respond to changes in the chemical compositions of their environment. In addition to CheY, the *N*-hydroxyarylamine *O*-acetyltransferase (NhoA) protein, the citrate synthase (Cs) and the isocitrate dehydrogenase (IcdH) enzymes, both of them belonging to the tricarboxylic acids cycle, and the adenosylmethionine synthase (MetK) are also substrates of CobB sirtuin (Zhang et al., 2013; Sun et al., 2016a; Venkat et al., 2018, 2019). Recently, the activity and stability of *E. coli* diguanylate cyclase DgcZ has also been demonstrated to be regulated by deacetylation catalyzed by CobB sirtuin (Xu et al., 2019). Furthermore, CobB regulates TCA cycle by delipoamidation PDH and KDH complexes (Rowland et al., 2017).

**FIGURE 1:**
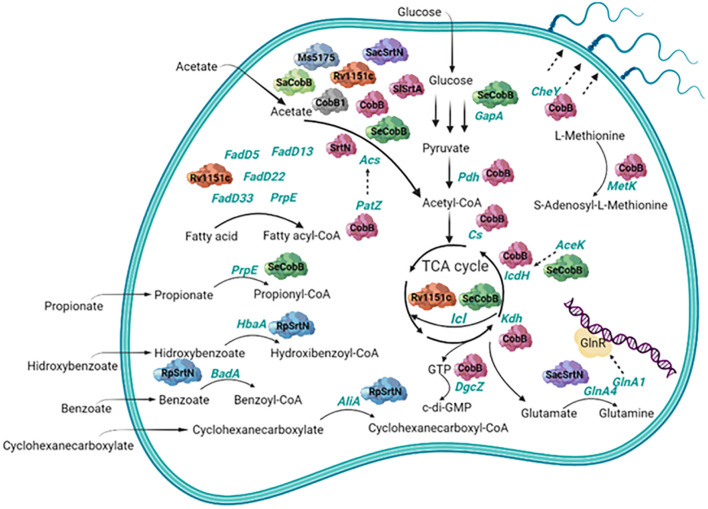
Scheme of the role of bacterial sirtuins in carbon and nitrogen metabolism. Deacetylated substrates are shown in green font and the bacterial sirtuins that deacetylate them have been represented as enzymes of different colors. Sirtuins shown here are represented with the abbreviated names and belong to different species. Correspondence with the full name and species for each one can be found throughout the text.

In 2016, nitrogen metabolism of *Saccharopolyspora erythraea (S. erythraea*) was shown to be regulated by sirtuin-dependent reversible lysine acetylation of two glutamine synthetases, GlnA1 and GlnA4. While acetylation inactivated GlnA4, acetylation of GlnA1 led to a fine modulation of the nitrogen regulator GlnR binding to DNA with a chaperone-like activity. Moreover, GlnR regulates transcription of AcuA acetyltransferase and of SacSrtN sirtuin, responsible for GlnA1 and GlnA4 acetylation and deacetylation, respectively, forming an autofeedback loop in nitrogen metabolism. This function of the acetylated GlnA1 over GlnR regulator seems to be conserved through actinomycetes such as *S. coelicolor* and *M. smegmatis* (You et al., 2016). GlnR also promotes transcription of the three AMP forming acetyl-CoA synthetases from *S. erythraea: acsA1*, *acsA2*, and *acsA3* in response to nitrogen availability. Their activity is regulated by lysine acetylation catalyzed by the acetyltransferase AcuA and the sirtuin SacSrtN (You et al., 2014, 2017). A more recent study also highlights the role of the SacSrtN sirtuin beyond GlnA1 and GlnA4 deacetylation. It suggests a central role of SacSrtN in the regulation of the primary and secondary metabolism in *S. erythraea* (Zhou et al., 2018). Role of bacterial sirtuins in carbon and nitrogen metabolism is resumed in Figure 1.

## Sirtuins in Bacterial Virulence and Resistance to Stress

*Salmonella enterica* is a facultative pathogen that survives within the cells of its host. TacT is a toxin acetyltransferase identified in *S. enterica* and responsible for antitoxin TacA acetylation, which is deacetylated by SeCobB sirtuin (VanDrisse et al., 2017). TacA acetylation increases TacT activity and therefore the persistence state inside a host. The two-component system PhoP-PhoQ is also involved in *S. enterica* virulence. Its activity is regulated by acetylation at K201 reverted by sirtuin SeCobB (Ren et al., 2016). Results showed that deacetylation of PhoP at K201 is essential for *Salmonella* pathogenesis. These data support the role of acetylation/deacetylation by SeCobB as a key regulator in *S. enterica* virulence (Yu et al., 2016). Reversible acetylation has also been related to *S. enterica* and *E. coli* survival under acid stress through modulation of Pat and CobB activity (Ma and Wood, 2011; Ren et al., 2015).

Role of sirtuin in *Yersinia pestis* (*Y. pestis*), responsible for causing pulmonary, bubonic and also septicemic plague in humans, has also been studied. Thus, *Y. pestis* reversible acetylation catalyzed by Pat acetyltransferase and CobB-like sirtuin, YpCobB, regulates the response to stress and virulence through PhoP acetylation and deacetylation (Liu et al., 2018). This recent finding should be studied in depth due to the important consequences that it may have on the development of drugs against this disease.

*Mycobacterium tuberculosis* is a pathogenic bacterium responsible for tuberculosis disease. *M. tuberculosis* is nonetheless present in almost 30% of the population of the world, although most of them do not develop the disease. A proteomic study carried out by Liu et al. (2014b) showed that lysine deacetylation by Rv1151c, the only sirtuin present in *M. tuberculosis*, regulated colony morphology, biofilm formation and resistance to heat stress. Interestingly, a strain of *M. smegmatis* with SrtN overexpressed showed a higher resistance to isoniazid, an anti-tuberculosis drug (Gu et al., 2015). INH is supplied as a prodrug that needs to be activated by the catalase peroxidase KatG to couple with NAD^+^ and inhibit the synthesis of mycolic acid, required for *M. tuberculosis* cell wall. SrtN overexpression increases *M. tuberculosis* resistance to INH through KatG down-regulation and intracellular NAD^+^ availability decrease.

## Sirtuins in Transcription and Translation Processes

Regulation of transcription by reversible lysine acetylation has been widely studied in eukaryotes and archaea but not in bacteria. After the first bacterial acetylome studies, several transcription factors were suggested to be regulated by acetylation (Yu et al., 2008; Zhang et al., 2009). *E. coli* RcsB, a transcription factor involved in cell division and flagellum synthesis, was the first transcription factor identified as CobB substrate in bacteria (Thao et al., 2010). RcsB DNA binding is regulated by the reversible acetylation of the conserved K180 (Thao et al., 2010). K154 was also identified as a target of acetylation and deacetylation by CobB *in vivo*. K154 acetylation inhibited RcsB while deacetylation by CobB activated it, regulating flagella biosynthesis and motility (Hu et al., 2013; Castaño-Cerezo et al., 2014). The global transcription factor cAMP receptor protein (CRP) has also been reported to be regulated by chemical acetylation, acetylation promoting activity in Class II promoters and ensuring adequate CRP steady state levels in *E. coli*, although CobB deacetylation has not been demonstrated yet (Écija-Conesa et al., 2020). DnaA is an initiator of replication that plays an essential role in the cell cycle. A recent study has revealed that the binding of *E. coli* DnaA to OriC is regulated by acetylation of the two lysines K178 and K243. Deacetylation of DnaA K243 by CobB is necessary for a correct DNA binding (Zhang et al., 2016; Li et al., 2017). A proteomic study carried out recently has identified the global anaerobic regulator FnrL from *Rhodobacter sphaeroides* (*R. sphaeroides*) as a substrate of its CobB sirtuin homolog, RsCobB. Thus, acetylation of FnrL decreases its DNA binding ability while the transcription of genes downstream of FnrL increases with deacetylation by RsCobB. This result suggests that reversible lysine acetylation might regulate anaerobic photosynthetic metabolism in this microorganism (Wei et al., 2017).

The first global analysis of the *E. coli* acetylome also revealed that RNA polymerase can be modified by acetylation. Shortly after, Lime et al. showed that the α-subunit of RNAP was regulated by acetylation and deacetylation of its K298. This is specifically required for the stress-responsive *cpxP* gene transcription (Lima et al., 2011). Gu et al. (2015) identified 27 proteins with an increased level of acetylation in a strain of *M. smegmatis* with the *srtN* gene deleted. These proteins included the beta subunit of RNA polymerase (rpoC) in addition to metabolic and ribosomal proteins. Other DNA-RNA related proteins have also been characterized as sirtuin substrates. Some examples are the proteins Ku and LigD, components of the non-homologous end-joining (NHEJ) system from *M. smegmatis* and *M. tuberculosis* or the *E. coli* RNAse II (Li et al., 2011; Song et al., 2016). Finally, sirtuins have also been related to translation through *E. coli* alanyl-tRNA synthetase and *Lactobacillus paracasei* ribosomal S4 30S protein deacetylation (Atarashi et al., 2016; Torres-Barredo et al., 2018; Umehara et al., 2018). The elongation factor Tu from *B. subtilis*, TufA, is also regulated by reversible acetylation. Although deacetylation of K42 TufA was catalyzed by the sirtuin SrtN, AcuC Zn-dependent deacetylase also deacetylated TufA, with even greater efficiency (Suzuki et al., 2019).

Sirtuins have emerged as important components of all regulation systems in bacteria, being involved in central metabolism, transcription or virulence regulation. However, the study of the role of sirtuins in bacteria has been limited to a few model organisms, leaving many species unstudied. In addition, even in these model organisms, the degree of knowledge that has been reached is very low, in comparison to eukaryotes. Their functional annotation is limited and further work need to be done to fill in this important gap. Bacterial sirtuins substrates characterized to date are shown in Table 1.

**TABLE 1:** Bacterial sirtuins characterized substrates.

Bacteria	Sirtuin	Substrate	Pathway	Reverted modification	References
*Salmonella enterica*	SeCobB	Acs	Acetate metabolism	Acetylation	Starai et al., 2002
	SeCobB	PrpE	Propanoate Degradation	Propionylation	Starai et al., 2003
	SeCobB	GapA	Glycolytic process	Acetylation	Wang et al., 2010
	SeCobB	Icl	Glyoxylate cycle	Acetylation	Wang et al., 2010
	SeCobB	AceK	Glycolytic process/glyoxylate cycle	Acetylation	Wang et al., 2010
	SeCobB	PhoP	Transcriptional regulatory protein	Acetylation	Ren et al., 2016
	SeCobB	TacA	Transport	Acetylation	VanDrisse et al., 2017
*Escherichia coli*	CobB	Acs	Acetate metabolism	Acetylation	Gallego-Jara et al., 2017
	CobB	CheY	Chemotaxis	Acetylation	Li et al., 2010
	CobB	Cs	Tricarboxylic acid cycle	Acetylation	Venkat et al., 2019
	CobB	IcdH	Tricarboxylic acid cycle	Acetylation	Venkat et al., 2018
	CobB	NhoA	Post-translational modification	Acetylation	Zhang et al., 2013
	CobB	RNAse II	RNA metabolism	Acetylation	Song et al., 2016
	CobB	MetK	Aspartate superpathway	Acetylation	Sun et al. (2016a)
	CobB	Alanyl-tRNA synthetase	Translation	Acetylation	Umehara et al., 2018
	CobB	RcsB	Transcription	Acetylation	Thao et al. (2010), Castaño-Cerezo et al., 2014
	CobB	RNAP	Transcription	Acetylation	Lima et al., 2011
	CobB	DNAa	Replication	Acetylation	Zhang et al., 2016
	CobB	PatZ	Post-translational modification	Acetylation	De Diego Puente et al., 2015
	CobB	PDH	Glycolytic process	Lipoamidation	Rowland et al., 2017
	CobB	KGH	Tricarboxylic acid cycle	Lipoamidation	Rowland et al., 2017
	CobB	DgcZ	Purine metabolism	Acetylation	Xu et al., 2019
*Bacillus subtilis*	SrtN	AcsA	Acetate metabolism	Acetylation	Gardner and Escalante-Semerena (2009)
	SrtN	TufA	Translation	Acetylation	Suzuki et al., 2019
*Mycobacterium tuberculosis*	Rv1151c	Acs	Acetate metabolism	Acetylation	Lee et al., 2012
	Rv1151c	FadD22, FadD2, FadD5, FadD13 and FadD33	Fatty acids metabolism	Acetylation	Nambi et al. (2013), Vergnolle et al., 2013
	Rv1151c	Icl	Glyoxylate cycle	Acetylation	Bi et al., 2017
	Rv1151c	Ku	NHEJ	Acetylation	Li et al., 2011
	Rv1151c	LigD	NHEJ	Acetylation	Li et al., 2011
*Mycobacterium smegmatis*	Ms5175	Ku	NHEJ	Acetylation	Li et al., 2011
	Ms5175	LigD	NHEJ	Acetylation	Li et al., 2011
	Ms5175	Acs	Acetate metabolism	Acetylation	Hayden et al., 2013
*Yersinia pestis*	YpCobB	PhoP	Transcriptional regulatory protein	Acetylation	Liu et al., 2018
*Lactobacillus paracasei*	LpSirA	S4 30S ribosomal protein	Translation	Acetylation	Atarashi et al., 2016
*Saccharopolyspora erythraea*	SacSrtN	GlnA1	Nitrogen metabolism	Acetylation	You et al., 2016
	SacSrtN	GlnA4	Nitrogen metabolism	Acetylation	You et al., 2016
	SacSrtN	AcsA1, AcsA2, AcsA3	Acetate metabolism	Acetylation	You et al., 2017
*Rhodobacter sphaeroides*	RsCobB	FnlR	Anaerobic respiration	Acetylation	Wei et al., 2017
*Rhodopseudomonas palustris*	RpSrtN	BadA, HbaA and AliA synthetases	Aromatic metabolism	Acetylation	Crosby et al., 2012
*Staphylococcus aureus*	SaCobB	Acs	Acetate metabolism	Acetylation	Burckhardt et al., 2019
*Streptomyces lividans*	SlSrtA	Acs and Aacs	Acetate metabolism	Acetylation	Tucker and Escalante-Semerena (2013), VanDrisse and Escalante-Semerena (2018)
*Streptomyces coelicolor*	CobB1	AcsA	Acetate metabolism	Acetylation	Mikulik et al., 2012

*The metabolic role and reverted modification catalyzed by sirtuins is shown.*

## Regulation of Bacterial Sirtuins

The global role demonstrated for bacterial sirtuins suggests a deep regulation of its expression and deacetylase activity at both the transcriptional and post-translational levels. This multidirectional regulation has been demonstrated in human sirtuins, but it is still mostly unknown in bacteria.

Eukaryotic sirtuins exist frequently in different isoforms as a consequence of alternative splicing (Zhang et al., 2021). In 2010, evidence of the existence of sirtuin isoforms in *S. enterica* were reported. Two active CobB isoforms, a shorter (236 amino acids) and a longer (273 amino acids) ones were studied. Moreover, transcription of the two isoforms started at two different sites (Tucker and Escalante-Semerena, 2010). In *E. coli*, the existence of these two isoforms has been also demonstrated and it has been suggested that they might be conserved in *Enterobacteriae* members, excepting species of the *Erwinia* genus (Umehara et al., 2018).

Regarding the transcriptional regulation of bacterial sirtuins, it has been reported that IolR protein, a regulator of the myo-inositol operon, positively controls the transcription of *cobB* in *S. enterica*, in addition to the acetyltransferase *pat* and *acs* genes (Hentchel et al., 2015). The regulation of sirtuins by different PTMs has been well covered and reported in eukaryotes (Flick and Lüscher, 2012). However, little is known about bacterial sirtuins PTMs. In 2016, a cross-talk between acetylation and phosphorylation was suggested in *M. smegmatis* and *tuberculosis*. Mycobacterial sirtuin deacetylase activity seems to be regulated by phosphorylation of a conserved threonine, which is, to date, the only regulation known of a bacterial sirtuin by phosphorylation (Yadav et al., 2016). Also in *M. smegmatis*, MSMEG_4620 sirtuin has been reported to catalyze its own ADP-ribosylation (Tan et al., 2015). More recently, regulation of the long isoform of CobB by N-terminal acetylation has been reported and negatively affects its deacetylase activity. Moreover, YiaC, a putative *S. enterica* acetyltransferase has been identified as the responsible for CobB N-terminal acetylation (Parks and Escalante-Semerena, 2020).

Nicotinamide adenine dinucleotide (NAD^+^) metabolism is closely related to sirtuins through NAD^+^ consumption and NAM production. Sirtuins activity inhibition by NAM is a global regulation mechanism strongly established (Jackson et al., 2003). The first kinetic study of a bacterial sirtuin was carried out in 2017, and the regulation of CobB *in vivo* by NAM concentrations was also demonstrated in our work (Gallego-Jara et al., 2017). To identify some *E. coli* CobB regulators, a study consisting in a proteome microarray identified, in 2014, 183 proteins that bound to CobB (Liu et al., 2014a). A more recent interactome study has revealed that phosphoribosyl pyrophosphate (PRPP) synthetase Prs protein, which was also identified as a potential interactor in 2014, increases CobB deacetylase activity and partially disable its inhibition by nicotinamide (Walter et al., 2020). Prs, is an enzyme responsible for PRPP synthesis, a pivotal metabolite involved, among others, in the NAD^+^ salvage pathway and NAD^+^ *de novo* biosynthesis. Prs regulation of CobB, might be the nexus between protein acetylation and NAD^+^ metabolism. The ubiquitous regulator cyclic diguanylate, c-di-GMP, also regulates negatively *E. coli* CobB, inhibiting its deacetylase activity. Moreover, CobB regulates the diguanylate cyclase enzyme, DgcZ, which produces c-di-GMP. CobB deacetylation regulates positively DgcZ, thus increasing DgcZ activity and c-di-GMP level, and establishing a regulator feedback loop (Xu et al., 2019).

## Phyletic Diversity

According to Table 1, substrates of 13 bacterial sirtuins (corresponding to 13 species) have been *in vitro* identified. These organisms represent only three bacterial phyla (Actinobacteria, Firmicutes, and Proteobacteria) and four bacterial classes (Actinomycetia, Bacilli, Alpha-, and Gamma-Proteobacteria) from the 166 phyla and 110 classes defined in the latest releases of the NCBI Taxonomy database (Agarwala et al., 2018). The Proteobacteria class is the most abundant in experimental data and number of species currently sequenced, containing the experimental model species *Escherichia coli*.

**FIGURE 2:**
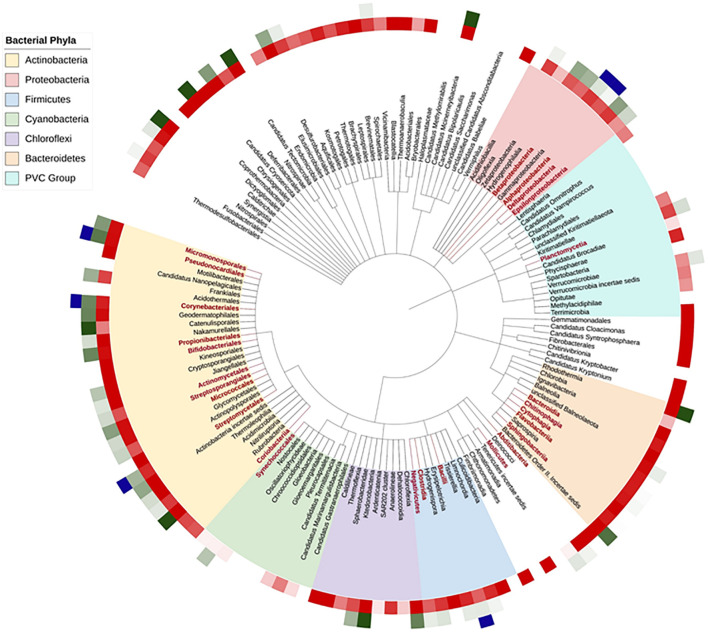
Sirtuins by bacterial class taxon. Bacterial classes are shown on a bacterial Tree of Life (Generated with PhyloT). Only those classes with more than 10 species genomes annotated in Uniprot are shown; those with more than 50 genomes are shown with branch and label in red. Bacterial phyla are indicated in the onset legend. The first layer indicates, in a red darkness gradient, the abundance of sirtuin-containing species per class. The second layer, in a green darkness gradient, indicates the abundance of species containing more than one sirtuin. The third layer indicates the classes with species that possess sirtuins that have been shown to regulate some substrate *in vitro.*

To gain a wider understanding of the diversity of the sirtuin distribution among the full range of phyla in the bacteria superkingdom, we conducted a bioinformatic analysis of the more than 8000 bacterial representative proteomes provided by Uniprot (Bateman et al., 2021). The Uniprot reference proteomes database covers fully annotated proteomes experimentally determined including at least one member of every bacterial phylum in the taxonomic tree (Figure 2). We determined the number of sirtuins encoded in each of the bacterial species searching for the Hidden Markov Model (HMM) profile that corresponds to the sirtuin domain family as defined in the Pfam domain database (Mistry et al., 2021). This family is coded as SIR2, with the accession number PF02146. A custom-made Python script allowed us to automatize the search and let us localize the number of sirtuins (if any) encoded in each of these reference bacterial genomes. The first layer of Figure 2 shows, in a red darkness gradient, the proportion of sirtuin-containing species with respect to the total number of different species per class, white indicating the absence of sirtuins-containing species in the class. This result indicates that, although sirtuins are widely distributed in prokaryotes and eukaryotes, they are not necessary for the proper growth of some organisms. Interestingly, the sirtuin abundance is not homogeneously distributed among bacterial taxons. As we show on the taxonomic tree pruned to class level, Phyla like Actinobacteria or Firmicutes have a tendency to include at least one sirtuin in their genomes while the majority of the species from the Cyanobacteria/Melainabacteria group lack sirtuins. Moreover, organisms lacking sirtuins perhaps have one or more enzymes that carry out sirtuin roles in metabolism, virulence, or transcription. Finally, it is important to highlight that the number of species and genomes covered by the Uniprot reference proteomes for each class is very disparate. Therefore, for some classes, abundance is the result of hundreds of species average, while other classes are composed of a few species.

The second layer in Figure 2 shows, in a green darkness gradient, the average number of species in each bacterial class that have at least one sirtuin encoded in its genome, white indicating the species with a single sirtuin. Sirtuins appear mostly as a single unit in bacteria, although an important number of species encoded for two homologous sirtuins. Examples have been found of species containing three or even more sirtuins among the proteins in their proteomes. From the 8002 species analyzed, 2321 do not have a single sirtuin encoded (a 29%) while 4396 (more than half of the species) have only 1 sirtuin (55%) and 1132 (14%) have 2 and only 153 have more than 2. The existence of duplicity in the sirtuin content in single bacterial species has been demonstrated experimentally as has been commented for *M. smegmatis* in the sirtuins in the carbon and nitrogen metabolism section. There is important heterogeneity in the number of sirtuins encoded by bacterial species even among classes belonging to the same phylum. In Bacteroidetes for example, while almost all classes encode for only one sirtuin, Rhodothermia species show more than three on average. Similar examples of classes with a high number of sirtuins are Ardenticatenia in the Chloroflexi phylum or Nitriliruptoria and Catenulisporales in the Actinobacteria one. Curiously, *M. tuberculosis* and *M. smegmatis*, both of them belonging to Actinobacteria phylum, have one and two homologous sirtuins, respectively, and the extra sirtuin from *M. smegmatis*, similar to Sirt4, has been observed in environmental mycobacterial species but not in pathogenic species (Tan et al., 2015).

Finally, as we observe in Figure 2, the number of homologous sirtuins is not directly related to the taxonomy or evolution of the different species, as species more evolved in the taxonomic tree of life can have fewer sirtuins or even none of them than more ancient species.

## Classification of Bacterial Sirtuins

Based on their phylogenetic relationships, sirtuins are generally grouped into several classes (Frye, 2000; Greiss and Gartner, 2009; Vassilopoulos et al., 2011; Costantini et al., 2013). The majority of these are represented by sirtuins present in human and most vertebrates: SIRT1-SIRT7 (classes I, II, III, and IV). A fifth class (class U) includes sirtuins mainly from bacteria and archaea. The last class to date was proposed in 2015, identified in microbial pathogens, and termed SirTMs (Rack et al., 2015).

**FIGURE 3:**
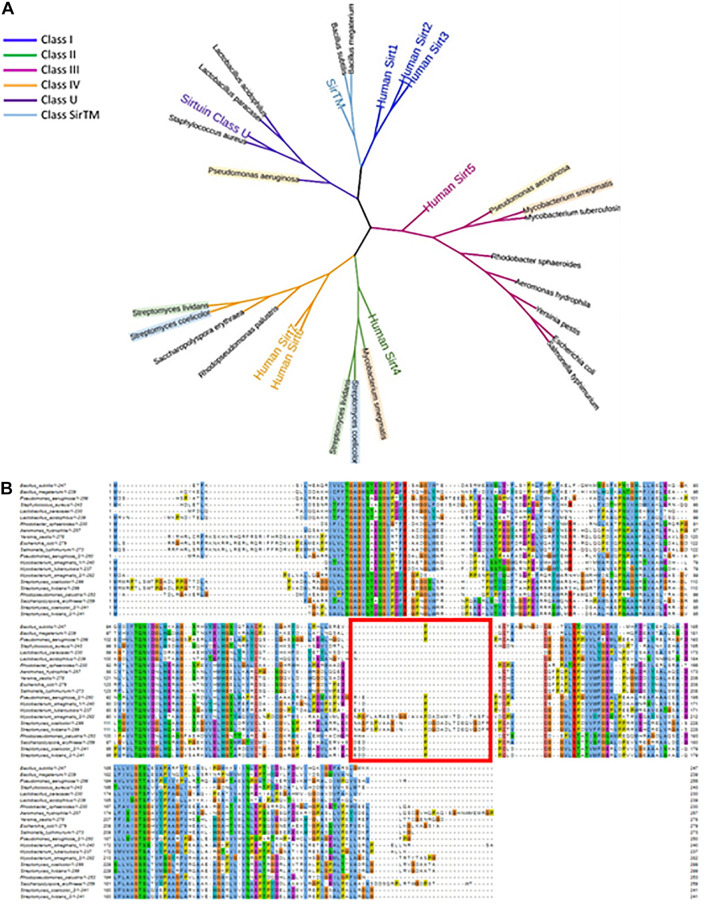
Phylogenetic tree and MSA of the sirtuins covered in this work. **(A)** Sirtuins are shown labeled with the name of their host species. Those species holding two sirtuins are labeled with a background color. The tree includes all human sirtuins and one representative of sirtuins class U and class TM for class identification. Branch colors indicate the sirtuins class. **(B)** Mafft multiple sequence alignment of the 21 sirtuins covered in this work. A red square indicates the loop insertion sequence.

To classify the sirtuins that have experimental evidence (summarized in Table 1), we generated a multiple sequence alignment [through the Mafft tool with an L-INS-I algorithm (Katoh et al., 2018)]. To the 13 bacterial sirtuins in Table 1, the homologous of these sirtuins were added. Furthermore, to increase the robustness of the analysis, sirtuins from *Lactobacillus acidophilus*, *Bacillus megaterium*, *Pseudomonas aeruginosa*, and *Aeromonas hydrophila* were also added. These bacteria were selected because the role of their sirtuins has been recently studied (Williams et al., 2012; Ouidir et al., 2015; Olesen et al., 2018; Wang et al., 2020). Altogether, the sequences of 21 sirtuins were aligned (Figure 3B). To assign the classes to the new sequences we included in the alignment a representative of each of the mentioned classes, including the seven human sirtuins. A phylogenetic tree was then built based on the multiple sequence alignment [FastTree with the JTT + CAT substitution model and a Shimodaira-Hasegawa test with 500 replicates for a bootstrap evaluation (Price et al., 2009; Gumerov and Zhulin, 2021)]. This allowed us to observe, even from such a limited set of sirtuins (only the ones with experimental evidence), that there are bacterial representatives for every class of sirtuin previously established from eukaryotes, except for class I (Figure 3A).

As shown in Figure 3A, most of the bacterial sirtuins with experimental evidence, and in particular those sirtuins belonging to the Proteobacteria phylum, have closer common ancestors with the human Sirt5 (class III) than with any other classes. However, there are examples of sirtuins more related to the Sirt4 (class II), with examples from the Actinobacteria phylum. Class IV is more widespread and is present mainly in Streptomyces and class U in Firmicutes. The most distant common ancestors from the bacterial sirtuins in our selection are those with the human sirtuins Sirt1, Sirt2, and Sirt3, i.e., those belonging to class I. However, sirtuins from the Bacilli genera have ancestors closer related to these class I components than to any other class, which makes a good example of the wide distribution in the classification of the bacterial sirtuins. Firmicutes phylum, to which Bacilli belong, appear in many phylogenetic trees as the older and most divergent group in Bacteria, which could explain the classification of some of their sirtuins in the most distant sirtuin class. Interestingly, bacterial sirtuins belong to different classes, since each of these sirtuin subtypes may be associated with different catalytic preferences as is already demonstrated in the human representative sirtuins. Human Sirt1-3 are considered strong deacetylases, while Sirt5 is mostly associated with other deacylase activities, such as desuccinylase, demalonylase and deglutarylase, and Sirt6 and Sirt4 show strong ADP-ribosyltransferase activity (Michishita et al., 2005; Du et al., 2011; Van Meter et al., 2011; Pannek et al., 2017). Moreover, Sirt6, and also Sirt1, Sirt2, Sirt3, and Sirt5, show deacylation activity against long-chain acyl groups and Sirt2 has lysine debenzoylation activity (Feldman et al., 2013; Huang et al., 2018). It is necessary to increase the number of bacterial sirtuins characterized *in vitro* to confirm whether this catalytic preference applies to bacterial counterparts. These observations suggest nevertheless a plausible specialization of sirtuins during evolution, allowing the species to adapt to the changing environmental conditions and availability of substrates through the diversification of the functional group they can transfer and the substrate they modify. Further studies on this direction are ongoing through the search for new substrates for sirtuins already characterized and the classification of new sirtuins in different bacterial species from more distant phyla.

Interestingly, those sirtuins that belong to the same species, in the cases where two are encoded, do not necessarily share the same sirtuin class. This is the case for the sirtuins in *Pseudomonas aeruginosa* (classes III and U), both *Streptomyces species* (classes II and IV), and *Mycobacterium smegmatis* (classes II and III). This observation indicates that the emergence of the paralogs in the species holding multiple sirtuin genes in their genomes is the result of duplication events that occurred in the common ancestors of the majority of the bacterial species summarized in this work. These duplications may have occurred therefore prior to the speciation. It is important to point out that none of the bacterial sirtuins representatives of the class I appear to have a paralog in the genomes of their species. This underscores the fact that this sirtuin class, which is the best studied in human and other vertebrates, is the furthest related with the majority of bacterial sirtuins, as any gene duplication has occurred in those cases posterior to a speciation and have therefore derived into a completely new species.

The results derived above from the classification of the bacterial sirtuins have to be taken with care, however, as they only include those species treated in the present work. Further work is to be done with a wider set of data that should include bacterial sirtuins from a greater number of bacterial species covering the full range of phyla and genera in the tree of life. The set extracted from only 18 species in this work has shown as a good representative of the bacterial sirtuins however and demonstrated that their class distribution is very wide.

Figure 3B shows the multiple sequence alignment that we originally generated for the construction of the phylogenetic tree. It contains the sequences of all the sirtuins treated in this work. It is interesting to point out that sequence lengths are very similar among all the bacterial sirtuins treated here. This is a fact that is not conserved in human or other vertebrate sirtuins, where there is a wide variety of lengths, especially in the C and N terminal fragments (Costantini et al., 2013). Human or other vertebrate sirtuins contain also a good number of isoforms that are related in a great extent to the C and N terminal sections (Szućko, 2016).

The main source of variation among the bacterial sirtuins herein studied comes from a 20–30 amino acid insertion in position 190 (related to *Escherichia coli*’s CobB sequence), that is included in one of the two sirtuins from *Mycobacterium smegmatis*, *Streptomyces coelicolor* and *Streptomyces lividans* (all three species from the Actinobacteria phylum, Figure 3B). This small insertion appears to be located between the helices α8 and α9 as shown in the sirtuin 3D structure (Cosgrove et al., 2006; Yuan and Marmorstein, 2012). These two regions are involved in the binding pocket of Zn^2+^, which plays a structural role. The loop insertion appears to be a common feature that is exclusive of class II sirtuins and has been previously observed in human Sirt4 (Pannek et al., 2017). It could play an important role in catalysis regulation, contributing to substrate binding and restricting active site dynamics. A larger investigation with the full set of reference proteomes from Uniprot is currently ongoing to further confirm the importance of this insertion and other features differentiating all bacterial sirtuins among them and with the known eukaryotic sirtuins.

## Conclusion

For years, sirtuins were only related to transcription regulation in eukaryotes by deacetylation of histones. Nowadays, it is known that they are involved in many metabolic pathways in addition to transcription regulation. Sirtuins have been widely studied in humans, where they regulate important metabolic pathways. However, little is known about sirtuins in lower organisms: only 13 bacterial sirtuins have been studied *in vitro* from the 8000 bacterial representative proteomes provided by Uniprot. With only this small sample a great diversity has been suggested both in the number of sirtuins per species and in the type of sirtuin. The phylogenetic study carried out in the last part of this review reveals the evolutionary differences between sirtuins of different bacterial species and even between homologous sirtuins. Future studies will be necessary to understand the diversity that emerged during evolution and thus to increase the knowledge about this important family of enzymes.

On the other hand, it is essential to continue investigating the stoichiometry of deacetylation by sirtuins to know if the low stoichiometry reported for *E. coli*’s CobB is conserved in other organisms and to know its physiological meaning. Furthermore, the low number of targeted proteins whose activity is regulated by sirtuins deacetylation identified to date contrasts to the high number of proteins that have been identified as their substrates in proteomic studies (Castaño-Cerezo et al., 2014; AbouElfetouh et al., 2015; Weinert et al., 2017). It seems difficult that the activity of all of them is regulated by sirtuin-mediated deacetylation, since the physiological behavior of the sirtuin-deficient strains is similar to that of the wild type. Some studies suggest that the sirtuins regulatory role might be more important in stressful or nutrient limited conditions, when acetylation, especially non-enzymatic is higher than in non-stressful situations (Schilling et al., 2015, 2019; Weinert et al., 2017).

Research in the field of bacterial sirtuins is essential to expand our knowledge about molecular signaling pathways, gene expression regulation, and associated physiological functions in prokaryotic organisms, which are fundamental for the advancement of fields such as synthetic biology or pharmacology.

## Author Contributions

JG-J, ÁO, and TD contributed to the conceptualization. JG-J and ÁO contributed to the writing—original draft preparation. JG-J, ÁO, GL, RS, MC, and TD contributed to the writing—review and editing. TD contributed to the project administration. TD contributed to the funding acquisition. All authors have read and agreed to the published version of the manuscript.

## Conflict of Interest

The authors declare that the research was conducted in the absence of any commercial or financial relationships that could be construed as a potential conflict of interest.

## Publisher’s Note

All claims expressed in this article are solely those of the authors and do not necessarily represent those of their affiliated organizations, or those of the publisher, the editors and the reviewers. Any product that may be evaluated in this article, or claim that may be made by its manufacturer, is not guaranteed or endorsed by the publisher.
